# Edible Coating Combining Liquid Smoke from Oil Palm Empty Fruit Bunches and Turmeric Extract to Prolong the Shelf Life of Mackerel

**DOI:** 10.3390/foods14010139

**Published:** 2025-01-06

**Authors:** Muhammad Faisal, Asri Gani, Murna Muzaifa, M. Bagas Heriansyah, Hera Desvita, Suraiya Kamaruzzaman, Ahmad Sauqi, Daru Ardiansa

**Affiliations:** 1 Department of Chemical Engineering, Faculty of Engineering, Universitas Syiah Kuala, Banda Aceh 23111, Indonesia; mfaisal@usk.ac.id (M.F.); asri_gani@usk.ac.id (A.G.); suraiya.k@usk.ac.id (S.K.); asauqi154@gmail.com (A.S.); daruardiansa@gmail.com (D.A.); 2 Climate Change Research Center, Universitas Syiah Kuala, Banda Aceh 23111, Indonesia; 3 Halal Research Center, Universitas Syiah Kuala, Banda Aceh 23111, Indonesia; 4 Oil Palm and Coconut Research Center, Universitas Syiah Kuala, Banda Aceh 23111, Indonesia; 5 Department of Agriculture Product Technology, Faculty of Agriculture, Universitas Syiah Kuala, Banda Aceh 23111, Indonesia; murnamuzaifa@usk.ac.id (M.M.); mbagasheriansyah@gmail.com (M.B.H.); 6 Research Center for Chemistry, National Research and Innovation Agency, B.J. Habibie Science and Techno Park, Serpong, South Tangerang 15314, Indonesia

**Keywords:** OPEFB, liquid smoke, turmeric, TVB-N, TPC, MPN, organoleptics

## Abstract

This research aimed to evaluate the use of edible coating from a combination of liquid smoke and turmeric extract as a preservative for mackerel at room temperature. Liquid smoke was obtained from the pyrolysis of oil palm empty fruit bunches (OPEFB) at a temperature of 380 °C and purified by distillation at 190 °C. Liquid smoke with a concentration of 3% was combined with turmeric extract at a ratio of 2, 4, 6, and 8 g/L (CLS 2:1, CLS 4:1, CLS 6:1 and CLS 8:1). TVB-N testing showed that the mixture of liquid smoke and turmeric at a ratio of CLS 6: 1 and CLS 8: 1 maintains the freshness of fish for 48 h. Meanwhile, organoleptic testing reports that the best mixture was CLS 8:1. The number of colonies in the CLS 2:1, CLS 4:1, CLS 6:1, and CLS 8:1 mixtures were 4.92, 4.92, 4.16, and 4 × 10⁵ colonies/g after 44 h of soaking. The MPN test result at 48 h of soaking is 1.1 × 10^3^ MPN/g. Generally, mackerel preserved with a mixture of turmeric extract and liquid smoke with a ratio of 8:1 can be consumed up to a shelf life of 48 h at room temperature storage.

## 1. Introduction

Fish is reported to contain high protein, vitamins, omega-3 polyunsaturated fatty acids, docosahexaenoic acid, eicosapentaenoic acid, and high nutritional value beneficial for body health [1,2]. This food ingredient is easily damaged since the meat is an ideal substrate for the life and growth of spoilage microorganisms, especially bacteria [3,4]. The water content in fish is high, around 65–80% [5], allowing possible biochemical reactions by enzymes in the body of fresh fish. The presence of biochemical reactions and oxidation causes tissue to decompose and produces chemical changes such as the texture of the meat becoming soft and the appearance of basic compounds in the protein causing a rancid odor. The presence of these volatile chemical compounds from the decomposition of fish meat provides a strong indication of a decline in quality. Therefore, these compounds are often used as an index and fish can last about 8 h after being caught [6,7].

To overcome the problem of quality decline in fish, various preservation methods have been developed, including those with natural ingredients [8]. An effective preservation method to extend the shelf life of fish is needed. People often use hazardous additives as a fish preservative, and this can have a negative impact on consumer health. The use of formalin has become a controversial issue and is prohibited in many countries due to the potential health risks. Several countries have implemented a ban on the use of the product to preserve fish. This compound is classified as a carcinogen and causes toxic effects in humans, such as irritation of the digestive tract, nausea, vomiting, and other digestive disorders. Therefore, the availability of safe and inexpensive natural preservatives is needed.

Natural preservatives used include red algae [9], peppermint essential oil [10], rosemary essential oil [11], clove essential oil [12], cinnamon oil [13], ‘Gabsi’ pomegranate peel extracts [14], terminalia ferdinandiana [15], thyme oil [16], curcumin [17], and liquid smoke [18]. Liquid smoke has bioactive compounds such as phenol, carbonyl, and organic acids functioning as antibacterials to maintain the quality of fish [19,20,21]. Liquid smoke can be produced from various biomass wastes such as coconut shells [22], terminalia cattapa wood [23], rice husks [24], and oil palm empty fruit bunches (OPEFB) [25]. However, the use as a preservative is still less popular because liquid smoke produces a fairly pungent odor. To reduce the odor, the concentration needs to be reduced and additional compounds from other natural ingredients such as turmeric extract are needed hence the antimicrobial properties are maintained.

Turmeric rhizome (*Curcuma domestical val*) is a biomaterial containing ingredients that can function as antioxidants and has received attention in food packaging [26,27]. This biomaterial is also widely used in herbal medicine and functional food, acting as anti-carcinogenic activities, antiangiogenesis, and antidiabetic [28,29,30,31]. Different bioactives are due to the presence of curcumin compounds [32]. Turmeric rhizomes can maintain fish quality due to curcumin compounds and essential oils. According to Pasaraeng et al. [33], the higher the concentration of curcumin, the lower the Total Volatile Base Nitrogen (TVB-N) value of fish. This shows that the inhibitory power of curcumin on bacterial growth is better. The combination of liquid smoke with turmeric, which contains active compounds such as curcumin, is expected to provide a synergistic effect in inhibiting the growth of microorganisms and slowing down the oxidation process in fish meat. This method is effective in extending shelf life as a sustainable solution for the fish processing industry. Therefore, this research aimed to evaluate natural preservatives from a combination of liquid smoke from OPEFB and curcumin extract to preserve mackerel. The ability of the natural preservatives was analyzed through Total Volatile Base (TVB) Testing, total plate count (TPC), most probable number (MPN), and organoleptic tests.

## 2. Materials and Methods

### 2.1. Materials

The materials used include OPEFB, turmeric extract, mackerel, distilled water (H_2_O), Sodium Hydroxide (NaOH; Merck, Darmstadt, Germany), Potassium Carbonate (K_2_CO_3_, Merck, Darmstadt, Germany), Trichloroacetate (TCA; Merck, Darmstadt, Germany), Ethanol (Merck, Darmstadt, Germany), Hydrochloric Acid (HCl; Merck, Darmstadt, Germany), Phenolptelain Indicator (PP; Merck, Darmstadt, Germany), Peptonwasser Buffering (BPW; Merck, Darmstadt, Germany), and Sodium Chloride (NaCl; Merck, Darmstadt, Germany).

### 2.2. Sample Preparation

OPEFB was obtained from a palm oil mill in Cot Girek village, North Aceh district, Aceh, Indonesia, and cut into 5–8 cm pieces and dried by being exposed to the sun for about three days until they were completely dry. Additionally, the 5% water content in the sample was tested. The turmeric was cleaned and dried to reduce the water content before grounding into powder.

### 2.3. Making Preservatives from a Combination of Liquid Smoke with Turmeric

**Figure 1:**
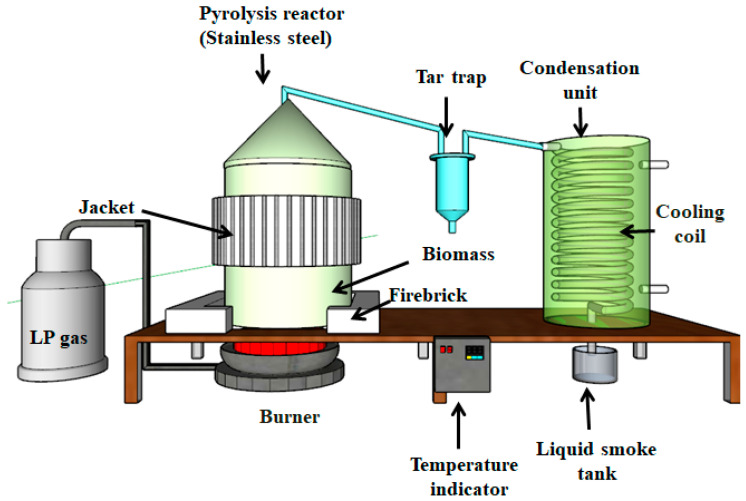
Schematic of the pyrolysis process.

A total of 3 kg of OPEFB was put into the reactor and pyrolyzed at a temperature of 380 °C. The resulting steam flowed to the condenser through a pipe connected to the reactor lid and produced condensate in the form of grade 3 liquid smoke and tar. The complete process of making liquid smoke was consistent with previous research [24], as reported in Figure 1.

The liquid smoke is purified using distillation at a temperature of 190 °C and diluted to 3% with distilled water. Furthermore, 3% (*v*/*v*) liquid smoke is added with turmeric powder at 2, 4, 6, and 8 g/L to obtain 4 combinations of CLS 2:1, CLS 4:1, CLS 6:1, and CLS 8:1, respectively.

### 2.4. Testing of a Mixture of Liquid Smoke with Curcumin on Mackerel Fish (Scomberomorus Commerson)

The test was conducted by dipping the mackerel into a mixture of liquid smoke with curcumin and analyzed every 4 h for 48 h. The mackerel (*Scomberomorus commerson*) samples were transferred to a sealed container and stored at room temperature during the preservation process. Mackerel that had not received any treatment were used as the control sample. The tests conducted were TVB-N followed the procedures outlined in SNI 2354.8:2009 [34]. The TVB-N value is calculated using the following formula:

$$TVBN=\frac{Vs-Vb\times NHCl\times 14.007\times 100}{Sample\;weight}$$

where TVB-N: Total Volatile Base (mgN/100 g), Vs: Sample volume (mL), Vb: Volume of solution without sample (mL). 

The TPC test was conducted in accordance with SNI 02-2725-1992 [35]. Nutrient Agar (NA) media in Petri dishes was used for the total plate count (TPC) test. The diluted mackerel sample was carefully transferred onto the surface of the NA media in the Petri dishes under aseptic conditions. After that, the plates were covered with plastic wrap and incubated at 37 °C for 24 to 48 h. A colony counter was used to count the bacterial colonies following incubation in order to calculate the total plate count.

The MPN test was carried out in following SNI 2897:2008 guidance [36]. The test consists of two phases: presumptive testing and confirmation testing, which confirms the results from the presumptive phase. A positive result is indicated by the production of gas or bubbles in the Durham tube.

The organoleptic tests such as Kamaruzzaman et al. [37]. Previous research calculated the average organoleptic value using the following equation [7].

(1) $$\begin{array}{l} AverageValue:x=\frac{\sum xi}{n} \end{array}$$

Description: x = average score, xi = organoleptic value of panelist i, n = number of panelists. Organoleptic testing involves using 37 panelist senses to test food, such as the hands to feel texture, the eyes to see color, and the nose to detect aroma. Seven of them were standard panelists, which are people who have knowledge and expertise in determining the grade of fish that is still fit for consumption, as well as good aptitude and sensitivity to product quality. The remaining thirty panelists were non-standard panelists, meaning they lacked the necessary training to perform organoleptic evaluations. The panelists were trained, educated, and given information on how to do the organoleptic evaluations prior to the test. To ascertain the degree of preference for mackerel fish preserved with different liquid smoke combinations and the duration of preservation, an organoleptic test was performed. Different ratios of liquid smoke mixture (CL 2:1, CL 4:1, CL 6:1, and 8:1) were used to soak the mackerel.

### 2.5. Statistical Analysis

All the experiments were performed with three replications. The data of the analyses were pooled, averaged, and standard deviation were calculated using MS-Excel software 2010.

## 3. Results

### 3.1. Total Volatile Base Nitrogen (TVB-N) Test

**Figure 2:**
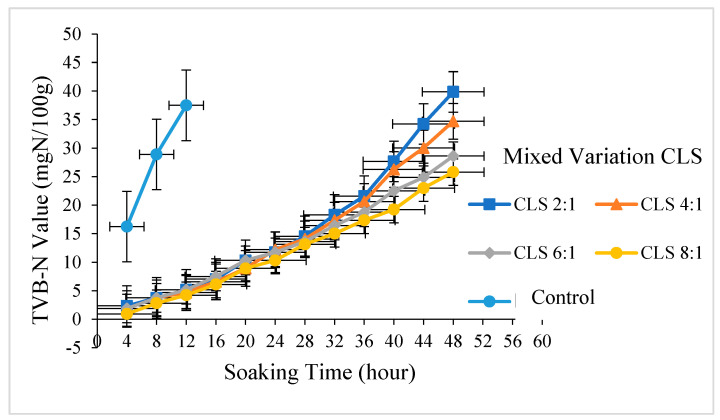
TVB-N value in mackerel fish samples coated with different concentrations of liquid smoke and turmeric.

TVB-N value is an important parameter used to determine the quality of food ingredients. In this context, a food ingredient will be considered unfit to eat with a TVB-N value that exceeds the acceptance limit. Pearson [38] stated that the acceptance limit for TVB-N value was 20–30 mg N/100 g, as reported in Figure 2.

The TVB-N values of mackerel fish shown in Figure 2 on the first day in each mixture were 11.725, 12.194, 11.725, and 10.318 mgN/100 g. These values show that at the beginning of storage, the fish was categorized as fresh with a TVB-N value range of 10–20 mgN/100 g [39]. The addition of liquid smoke from OPEFB with a combination of turmeric can reduce TVB-N value in mackerel fish compared to control samples. The TVB-N value after soaking for 8 h was 28.901 mgN/100 g without preservative treatment from a mixture of liquid smoke with turmeric. This value is close to the maximum TVB-N value which is not good for consumption. Meanwhile, the TVB-N value obtained was 37.501 mgN/100 g at 12 h. Fish soaked using liquid smoke and CLS in 2:1 and 8:1 mixtures of turmeric and liquid smoke lasted for 40 and 48 h, respectively.

The TVB-N value continues to increase during storage time, indicating a decline in the quality of mackerel. The soft texture and high protein content of fish causes protein degradation, peptide compounds, and amino acid content to produce volatile base compounds [40]. Bekhit et al. [41] stated that the degradation of enzymes produced volatile base compounds. The TVB-N value is influenced by the amount of non-protein nitrogen in the fish [42]. The highest value of mackerel in the CLS 2:1 sample was obtained with a TVB-N value of 39.86 mgN/100 g at a storage time of 48 h, and the value exceeded the limit for consumption. Fadhli et al. [39] stated that fish were included in the fresh category with a maximum TVB-N value of 30 mgN/100 g. According to Izza et al. [43], the TVB-N value of tofu preserved using liquid smoke derived from teak and pine wood indicates a shelf life of 4 days, maintaining 24.51 mgN/100 g. However, the TVB- value exceeds the safe consumption limit (>35 mgN/100 g) on day 5. The presence of curcumin compounds in turmeric can inhibit the growth of bacteria damaging fish meat [44]. Therefore, the preservative from CLS preserves mackerel for 48 h of storage.

### 3.2. Organoleptic Test

#### 3.2.1. Taste

**Figure 3:**
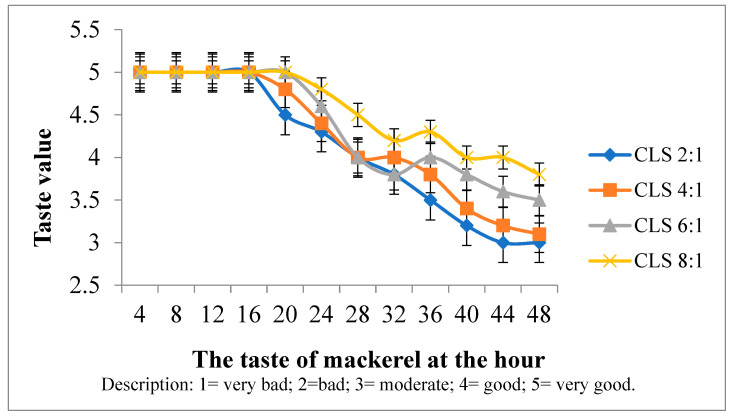
Taste testing of preserved mackerel in various CLS.

Sensory evaluation of preserved mackerel, treated with a combination of liquid smoke and curcumin, was conducted through taste testing by a panel of assessors. Before testing, the mackerel was steamed for 10 min, as reported in Figure 3.

Figure 3 shows that the addition of liquid smoke with a high concentration in preservation can slow down the aroma and taste of mackerel and last up to 30 h. In terms of taste, fish with CLS variations of 20 h of immersion are still delicious. However, the best is CLS 8:1 mixture as reported by the different variations. This mixture has a delicious taste compared to others at a soaking time of 48 h because the concentration of curcumin inhibits bacterial activity. Therefore, the taste is delicious and the addition of liquid smoke to the soaking water causes the fish to be dominated by a slightly smoky taste. The taste changes faster in less than 12 h when compared to mackerel. For CLS 8:1 at 20 h, the percentage of panelist assessment was 100% by giving a score of 5. At 48 h, the panelists gave more scores of 4, resulting in a percentage of 68%, while others reported 3. The results are appropriate to Elshehawy and Farag [45], where smoked chicken with 1% and 2% received higher acceptance and value for taste, texture, color, and aroma. Faisal et al. [7] showed that 2–3% liquid smoke had a taste acceptable to the panelists for 48 h of preservation time.

#### 3.2.2. Aroma

**Figure 4:**
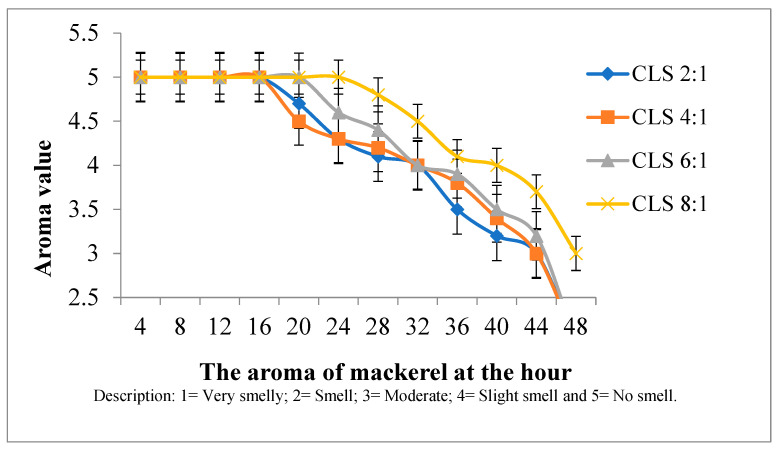
Aroma testing on preserved mackerel in various CLS.

Aroma testing was conducted using the nose of each panelist. Meanwhile, the fish was steamed for 10 min before testing. In terms of aroma in mackerel (Figure 4) with CLS 2:1 and CLS 4:1 mixtures, the fish still had a good aroma at 20 h of soaking time. In contrast to the CLS 8:1 and CLS 6:1 mixtures of 32 h, the aroma of the fish still tasted good. After 10 h without treatment with the CLS preservative mixture, the aroma of the mackerel developed signs of spoilage. The smoky aroma produced was absorbed into the fish layer. The aroma became pungent due to the gradual reduction in acetic acid content. An unpleasant aroma could also be used as an indication of product damage caused by an oxidation reaction. Additionally, the occurrence of fat oxidation leads to an undesirable odor in fish [46]. In comparison to other variations, CLS 8:1 mixture showed an improved profile due to the ability to increase the amount of turmeric and increase the odor associated with liquid smoke. This is caused by the substances contained in turmeric, namely curcumin content which gives a distinctive aroma to the preservative mixture [47]. The preference value for preserved mackerel in the CLS 8:1 variation with a soaking time of 32 h was 65% at a score of 5, while others were 3 and 4. Similar results with the use of 3% liquid smoke from durian skin can still be accepted at 48 h [7].

#### 3.2.3. Texture

**Figure 5:**
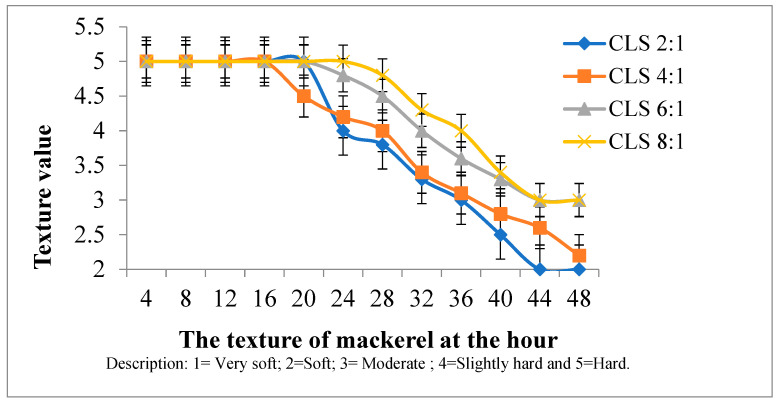
Texture testing on preserved mackerel in various CLS.

Based on Figure 5, the best texture of mackerel is in fish with the addition of liquid smoke in CLS 8:1 and CLS 6:1 mixture variations. In the CLS 2:1 and CLS 4:1 variations, the texture of the fish begins to change to soft at 32 h. Generally, the texture of the fish soaked in water without preservatives begins to change at 10 h because of increased bacterial growth. Fish experience a decline in quality when the texture of the meat becomes soft due to the enzymatic process in muscle tissue, such as cathepsin and collagenase. The cathepsin enzyme causes the texture to become soft due to protein degradation, while the collagenase breaks down polypeptide bonds [48]. For the presentation of preference at CLS 8:1 with a time of 32 h, 73% of panelists gave a score of 4 while others scored 3 and 5. At 48 h, the score given by all panelists was 3. According to Syarif et al. [49], pyrolysis of ironwood at a temperature of 400 °C produced liquid smoke used to preserve mackerel. This was achieved by using 5% liquid smoke to maintain a shelf life of 3 days with a texture value lower than fresh fish.

#### 3.2.4. Color

**Figure 6:**
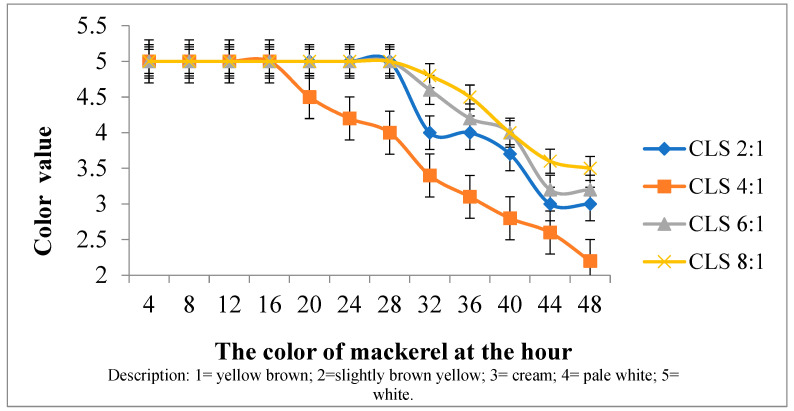
Color testing on preserved mackerel in various CLS.

The color evaluation of the mackerel in the organoleptic test was based on the color of its flesh. Figure 6 describes the color score values utilized in the organoleptic test.

The mackerel soaked with various mixtures of liquid smoke and turmeric gave different color change effects to the soaking time (Figure 6). According to Joesidawati [50], carbonyl compounds (aldehydes and ketones) have a major influence because the product changes due to the interaction between carbonyl and amino groups, and the color of the fish remains white. The rate at which the turmeric is absorbed into the fish tissue or the way the turmeric extract reacts with the other chemicals in the liquid smoke mixture. Turmeric’s structure is changed throughout the extraction and purification processes, making it incapable of being absorbed [51]. In the variation in CLS mixture 8:1, the color is yellowish due to the influence of high turmeric. Meanwhile, the color of the fish soaked without CLS mixture began to change to brownish at 12 h of soaking. A type of food product with high nutritional value, taste, and texture but without good color can reduce the demand of consumers. The preference value for mackerel at CLS 8:1 was 65%, giving a score of 3. The use of liquid smoke from durian skin with a concentration of 0.5–3% maintains the value during storage and the color begins to change after 42 h due to the high content of phenol and acetic acid [7]. The intensity of the change in the sample is due to the Maillard reaction occurring between the carbonyl and amino acid group or protein [52].

### 3.3. Total Plate Count (TPC) Test

The bacterial content in a product is among the microbiological parameters in determining the suitability of a product for consumption [53]. In this context, the microbial contamination of fishery products occurs during handling, distribution, as well as storage, and processing. Analysis of the number of bacteria determines the growth rate during storage and the results of the TPC analysis of mackerel are presented in Table 1.

The number of microbes increased from the initial state in the CLS 2:1 mixture, which was 1.2 × 10^5^ colonies/g to 1.88 × 10^5^ colonies/g. In this context, the microbes at 24 h immersion are in the safe consumption zone of 1 × 10^5^ colonies/g based on SNI 02-2725-1992 [35]. At 20 h to 28 h immersion time, the number of microbial colonies decreased to 2.08 × 10^5^ colonies/g, 1.88 × 10^5^ colonies/g and 1.6 × 10^5^ colonies/g. This is due to high bacteriostatic properties, affecting the reproducibility of bacteria [54]. According to Sasongko et al. [55] the average bacterial colony/g of smoked rabbit meat using immersion with 0% contained 27.4 × 10^5^ colonies/g of bacteria. Meanwhile, the treatment of rabbit meat soaked using 1% liquid smoke contained 18.4 × 10^5^ colonies/g of bacteria. This shows the activity of coconut shells in inhibiting and killing bacteria in smoked rabbit meat. Based on other research, the TPC value of fish soaked with liquid smoke with a concentration of 2.5% experienced the greatest microbial growth on the 4th day of storage of 3.1 × 10^5^ colonies/g. During storage until the 20th day, the TPC value of fish boiled using 2.5% liquid smoke experienced a decrease of 3.1 × 10^5^ colonies/g [56]. According to Xin et al. [57], soaking green mussels can reduce the TPC value from 3.65 to 3.03 log CFU/g. At the time of soaking 36 h, the number of microbes continues to increase with the combination of mixtures. The media supporting adaptation and environmental conditions suitable for microbial growth are important factors influencing the increase in microbes. However, the use of liquid smoke with a combination of turmeric on mackerel can inhibit bacterial growth due to the content of phenol, carbonyl, and acid as well as the derivatives [58].

### 3.4. Most Probable Number of Escherichia coli

MPN test on mackerel preservation against *Escherichia coli* bacteria can be seen in Table 2. Bacterial growth increased after 12 h of preservation, where the value was more than 0.3 MPN/g. The number of *E. coli* in mackerel with the treatment ranged from 3.0 MPN/g to 3.6 MPN/g at 4 h of observation and decreased to 0.3 MPN/g at 8 h. The presence of acid, phenol, and phenolic compounds in liquid smoke interferes with the growth of *E. coli.* Liquid smoke is a strong bactericide that can stop the growth of *E. coli* and other pathogens [59]. Previous research showed that smoked fish products given liquid smoke from sawdust could inhibit the growth of coliform or *E. coli* [60]. In addition, turmeric has antioxidant properties, which maintain fat stability and extend the shelf life of products [61]. The use of the extract can give a natural yellow color to mackerel which adds to the visual appeal. In the CLS2:1 treatment at a storage time of 32 h, the MPN value exceeded the maximum limit of microbial contamination at 1 × 10^5^ MPN/g [36]. Based on the observations, mackerel given liquid smoke with a higher concentration of turmeric showed better resistance to microbial contamination compared to those coated with a lower concentration of the extract. Each edible coating CLS 4:1, CLS6:1, and CLS8:1 can extend shelf life by 36, 40, and 48 h, respectively.

## 4. Conclusions

In conclusion, the ratio of liquid smoke and turmeric concentrations affected the preservation ability of edible coating. Meanwhile, TVB, TPC, and MPN values decreased with the increasing ratio. At 48 h of storage time and CLS 8:1, the TVB, TPC, and MPN values were 25.795 mgN/100 g, 4.96 × 10^5^ colonies/g, and 1.1 × 10^3^ MPN/g, respectively. This showed that the condition of the fish was still suitable for consumption within the permitted limit. For CLS 2:1, the TVB value increased to 39.865 mgN/100 g since the condition of the fish was no longer suitable for consumption. The results of the organoleptic test for taste on the CLS 8:1 edible coating showed that the fish lasted up to 48 h with a value of 3.8. For texture and aroma, the mixture of CLS 8:1 was quite good since the mackerel was in good condition. Therefore, a mixture of edible coating liquid smoke from OPEFB and turmeric could be an effective natural preservative to extend the shelf life of mackerel with suitable packing techniques like vacuum sealing or modified atmosphere packaging. Future research could be explored further in future studies.

## Figures and Tables

**Table 1:** Analysis data of total plate count test of mackerel with various CLS.

Storage Time (Hours)	Number of Colonies in CLS Variation (×10^5^ Colonies/g)			
	CLS 2:1	CLS 4:1	CLS 6:1	CLS 8:1
4	1.2	0.68	0.48	0.16
8	1.8	1.44	1.2	0.48
12	2.52	2.28	1.72	1.24
16	2.88	2.8	2.68	2.56
20	2.08	1.88	1.76	1.56
24	1.88	1.36	1.24	1.08
28	1.6	1.2	1.08	0.92
32	2.28	1.84	1.48	1.4
36	3.36	3.4	3.12	3.08
40	4.08	3.8	3.56	3.44
44	4.92	4.92	4.16	4
48	5.64	5.44	5.08	4.96

Note: CLS = Turmeric: liquid smoke combination (g/L).

**Table 2:** Most probable number of *E. coli* data of mackerel in various CLS.

Storage Time (Hours)	MPN/g			
	CLS 2:1	CLS 4:1	CLS 6:1	CLS 8:1
4	3.6 × 10^1^	3 × 10^1^	3 × 10^1^	3 × 10^1^
8	<0.3 × 10^1^	<0.3 × 10^1^	<0.3 × 10^1^	<0.3 × 10^1^
12	0.3 × 10^1^	0.3 × 10^1^	<0.3 × 10^1^	<0.3 × 10^1^
16	2.9 × 10^1^	1.5 × 10^1^	1.5 × 10^1^	0.7 × 10^1^
20	2.1 × 10^2^	2.7 × 10^1^	2.3 × 10^1^	1.4 × 10^1^
24	2.9 × 10^2^	2.8 × 10^1^	2.3 × 10^1^	1.2 × 10^2^
28	4.6 × 10^2^	1.6 × 10^2^	1.2 × 10^2^	1.5 × 10^2^
32	>1.1 × 10^3^	2.9 × 10^2^	1.5 × 10^2^	2.4 × 10^2^
36	>1.1 × 10^3^	1.1 × 10^3^	2.4 × 10^2^	2.9 × 10^2^
40	>1.1 × 10^3^	>1.1 × 10^3^	2.9 × 10^2^	4.6 × 10^2^
44	>1.1 × 10^3^	>1.1 × 10^3^	>1.1 × 10^3^	1.1 × 10^3^
48	>1.1 × 10^3^	>1.1 × 10^3^	>1.1 × 10^3^	1.1 × 10^3^

Note: CLS = Turmeric: liquid smoke combination (g/L).

## Data Availability

The original contributions presented in the study are included in the article, further inquiries can be directed to the corresponding author.
